# Antitumor activities of ATP-competitive inhibitors of mTOR in colon cancer cells

**DOI:** 10.1186/1471-2407-12-86

**Published:** 2012-03-08

**Authors:** Benjamin Blaser, Laurent Waselle, Anne Dormond-Meuwly, Marc Dufour, Didier Roulin, Nicolas Demartines, Olivier Dormond

**Affiliations:** 1 Department of Visceral Surgery, Centre Hospitalier Universitaire Vaudois and University of Lausanne, Pavillon 4, Av. de Beaumont, 1011 Lausanne, Switzerland

**Keywords:** Colon cancer, mTOR, Rapamycin, NVP-BEZ235, PP242, Proliferation, Xenograft

## Abstract

**Background:**

The mammalian target of rapamycin (mTOR) is frequently activated in colon cancers due to mutations in the phosphatidylinositol 3-kinase (PI3K) pathway. Targeting mTOR with allosteric inhibitors of mTOR such as rapamycin reduces colon cancer progression in several experimental models. Recently, a new class of mTOR inhibitors that act as ATP-competitive inhibitors of mTOR, has been developed. The effectiveness of these drugs in colon cancer cells has however not been fully characterized.

**Methods:**

LS174T, SW480 and DLD-1 colon cancer cell lines were treated with PP242 an ATP-competitive inhibitor of mTOR, NVP-BEZ235, a dual PI3K/mTOR inhibitor or rapamycin. Tumor cell growth, proliferation and survival were assessed by MTS assay, 5-bromo-2'-deoxyuridine (BrDU) incorporation or by quantification of DNA fragmentation respectively. *In vivo*, the anticancer activity of mTOR inhibitors was evaluated on nude mice bearing colon cancer xenografts.

**Results:**

PP242 and NVP-BEZ235 reduced the growth, proliferation and survival of LS174T and DLD-1 colon cancer cells more efficiently than rapamycin. Similarly, PP242 and NVP-BEZ235 also decreased significantly the proliferation and survival of SW480 cells which were resistant to the effects of rapamycin. *In vivo*, PP242 and NVP-BEZ235 reduced the growth of xenografts generated from LS174T and SW480 cells. Finally, we also observed that the efficacy of ATP-competitive inhibitors of mTOR was enhanced by U0126, a MEK inhibitor.

**Conclusions:**

Taken together, these results show that ATP-competitive inhibitors of mTOR are effective in blocking colon cancer cell growth *in vitro *and *in vivo *and thus represent a therapeutic option in colon cancer either alone or in combination with MEK inhibitors.

## Background

Colorectal cancer (CRC) is one of the leading cause of cancer-related deaths worldwide [1]. Over the last decade, new therapeutic options for the treatment of CRC have been developed including targeted therapies. For example, drugs that block the vascular endothelial growth factor or the epidermal growth factor receptor have shown clinical activities and have been approved for the treatment of CRC [2]. However, despite these new treatments, the prognosis of CRC remains poor and new therapeutic strategies still need to be explored.

The mammalian target of rapamycin (mTOR) is a serine/threonine kinase, present in two functionally distinct complexes mTORC1 and mTORC2. While mTORC1 is composed of mTOR, mLST8, raptor, deptor and PRAS40, mTORC2 consists of mTOR, rictor protor, mLST8, deptor and sin1 [3,4]. mTORC1 regulates cell growth by controlling mRNA translation initiation and progression by phosphorylating two well characterized downstream effectors: S6K1 and 4E-BP1 [5]. In addition, mTORC1 also regulates ribosome biogenesis, autophagy and lipid biosynthesis. mTORC2 is involved in cell survival and proliferation by phosphorylating members of the AGC kinase family including Akt, protein kinase C and serum-and glucocorticoid-regulated kinase [6-8]. Of note, whereas mTORC1 is sensitive to acute exposure to rapamycin, mTORC2 is not. However in a subset of cells, prolonged exposure to rapamycin also inhibits mTORC2 [9].

Emerging data have shown that mTOR is implicated in the progression of CRC and represents a promising target in the treatment of CRC. Indeed, components of mTOR signaling pathway are frequently activated or over-expressed in CRC [10,11]. For example, genetic aberrations of the catalytic subunit of the phosphatidylinositol 3-kinase (PI3K), an upstream effector of mTORC1 and mTORC2, are frequent in colon cancer [12,13].Moreover, the inhibition of mTOR signals by allosteric inhibitors such as rapamycin or small interfering RNA has been shown to reduce colon cancer growth in different experimental settings [10,11,14,15]. Recently, a new class of mTOR inhibitors have been developed that target the kinase domain of mTOR and referred as ATP-competitive inhibitors of mTOR [16,17]. In contrast to rapamycin which targets only certain functions of mTORC1, ATP-competitive inhibitors of mTOR inhibit both mTORC1 and mTORC2. Furthermore, a subset of these inhibitors also blocks PI3K in addition to inhibit mTORC1 and mTORC2 [18]. In this study, we have determined the anticancer activity of PP242 [19], a kinase inhibitor of mTOR and NVP-BEZ235 [20], a dual PI3K/mTOR inhibitor, in colon cancer cells, both *in vitro *and *in vivo*.

## Methods

### Cell lines, antibodies and reagents

The human colon cancer cell lines LS174T, DLD-1, SW480, SW620, HT29, Caco-2, and HCT-116 were maintained in Dulbecco's modified eagle's medium supplemented with 10% fetal calf serum. Antibodies directed against phospho-Akt (Ser473), Akt, phospho-S6 ribosomal protein (Ser235/236), S6 ribosomal protein and cleaved caspase-3 were from Cell signaling technology (Danvers, MA, USA). Rapamycin, U0126 and NVP-BEZ235 were from LC laboratories (Woburn, MA, USA). PP242 was from Chemdea (Ridgewood, NJ, USA). For *in vitro *experiments, all inhibitors were dissolved in dimethyl sulfoxide (DMSO).

### Western blot analysis

Western blot were performed as previously described [21].

### MTS proliferation assay

LS174T, SW480, DLD-1, Caco-2, HCT-116, SW620 and HT-29 cells were plated on 96-well plates (Costar) at 10'000 cells per well and cultured in DMEM 10% FBS. Twelve hours later, cells were treated with rapamycin (10 nM), NVP-BEZ235 (100 nM), PP242 (100 nM) or DMSO as a control. Cellular proliferation was monitored after 48 hours of treatment with the CellTiter 96^® ^Aqueous One Solution (Promega Corporation) colorimetric assay by following the manufacturer's instructions.

### BrDU incorporation assay

BrDU incorporation assay was performed as previously described [22].

### Cell survival studies

LS174T, SW480, DLD-1 cells were plated in 96-well plates at 30,000 cells per well. Twelve hours later, cells were treated with rapamycin (10 nM), NVP-BEZ235 (100 nM), PP242 (100 nM), either alone or in combination with U0126 (10 μM) for 48 hours. Subsequently cells were harvested and apoptosis was determined using the Cell Death Detection ELISA plus kit (Roche) and following the manufacturer's instructions. Results are represented as the mean enrichment factor (absorbance of the treated cells/absorbance of the control cells).

In addition, cell apoptosis was also quantified using flow cytometry. LS174T, SW480 and DLD-1 cells were plated in 6-well plates at 300 000 cells per well and treated as above. After 48 hours of treatment cells were collected and fixed in 70% ethanol for 24 hours. Cells were subsequently resuspended in phosphate buffered saline (PBS) containing 20 μg/ml propidium iodide and 200 μg/ml RNAse and incubated for 30 minutes at 37°C. The percentages of sub-G1 population were determined by flow cytometry.

### Tumor xenografts

Animal experiments were approved by the ethics committee of the cantonal veterinary office of Canton Vaud (Authorization 2047) and conducted in accordance with the regulations of the Service of Consumables and Veterinary Affairs-Division of Animal Protection (SCAV-EXPANIM). Female nude mice aged 8 weeks were purchased from Charles River (Charles River Laboratories, St. Germain sur l'Arbresle, France). One million LS174T or SW480 cells were injected subcutaneously into the flank of nude mice. Once the tumor xenografts reached 25 mm^3^, mice were randomized into different groups (n = 5 in each group). Mice were treated with rapamycin (1.5 mg/kg/d, i.p.), NVP-BEZ235 (30 mg/kg/d, p.o.), PP242 (60 mg/kg/d, p.o.) either alone or in combination with U0126 (40 μmol/kg/d, i.p.). All mice received both p.o. and i.p. doses of vehicle to control for morbidity associated with treatment. NVP-BEZ235 was solubilized in one volume of N-methylpyrrolidone and further diluted in nine volumes of PEG 300. PP242 was dissolved in PEG 300. Stock solutions of rapamycin and U0126 were prepared in DMSO and further diluted in PBS before injection. Tumor volumes were measured using caliper measurements every day and calculated with the formula V = π/(6a2b) where a is the short axis and b the long axis of the tumor. Animals were sacrificed after 20 days of treatment and the tumors were excised and processed for further analysis.

### Immunochemistry

Tumor xenografts were carefully removed and rapidly frozen in OCT compound (Tissue-Teck) on dry ice. Eight μm transverse sections were cut on a cryostat (CM 1850, Leica), and processed for immunolabeling with an anti-Ki-67 (Novocastra) as previously described [22]. Ki-67 positivity was quantified and expressed as % of cells positive for Ki-67/total number of cells (300 cells counted per tumor; five tumors in each group).

### Statistical analysis

Data were analyzed by Student's t-test or one way ANOVA. Values of *P *< 0.05 were considered statistically significant.

## Results

### Concentration-dependent effects of ATP-competitive inhibitors of mTOR on mTORC1 and mTORC2 activity in colon cancer cells

**Figure 1:**
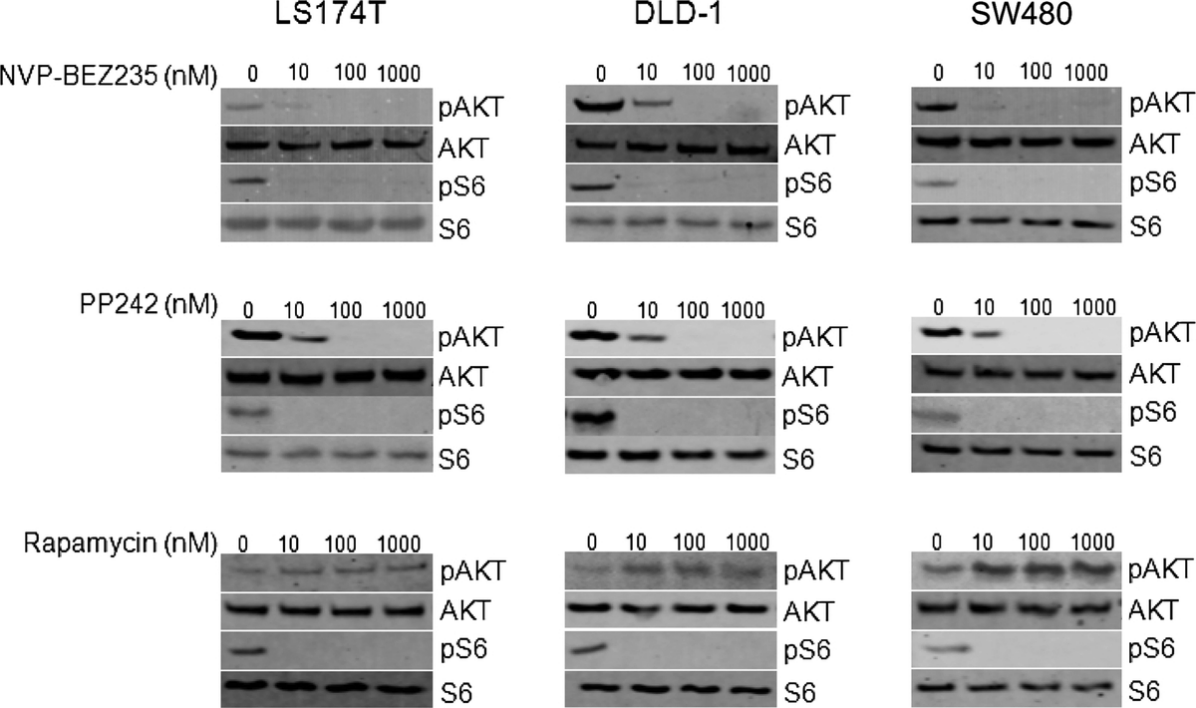
**Effects of rapamycin, NVP-BEZ235 and PP242 on mTORC1 and mTORC2 activity in colon cancer cells**. LS174T, DLD-1 and SW480 colon cancer cells were treated with increasing concentrations of rapamycin, NVP-BEZ235 or PP242 for six hours. Cells were lysed and analyzed by Western blot for phospho-S6 ribosomal protein and phospho-Akt as a read-out for mTORC1 and mTORC2 activity respectively. Total S6 ribosomal protein and total Akt were used as loading controls. The illustrated blots are representative of three independent experiments.

The activity of various inhibitors of mTOR was tested on colon cancer cells that harbor distinct mutations of the catalytic subunit of PI3K (PI3KCA) [23,24]. LS174T (*PI3KCA *mutation on exon 20), DLD-1 (*PI3KCA *mutation on exon 9) and SW480 (*PI3KCA *wild type) colon cancer cells were treated with increasing concentrations of rapamycin, PP242 [19], a specific mTOR inhibitor, or NVP-BEZ235 [20], a dual PI3K/mTOR inhibitor for six hours. Rapamycin, NVP-BEZ235 and PP242 inhibited mTORC1 activity at 10 nM as observed by the dephosphorylation of S6 ribosomal protein on Western blot analysis (Figure 1). At higher concentrations (100 nM), NVP-BEZ235 and PP242 also blocked mTORC2 activity as evidenced by the dephosphorylation of Akt (Figure 1). In contrast, rapamycin increased Akt phosphorylation consistent with the removal of a negative feedback loop whereby the inhibition of mTORC1 induces PI3K/Akt activation [25].

### Effect of ATP-competitive inhibitors of mTOR compared to rapamycin on colon cancer cell proliferation and survival

**Figure 2:**
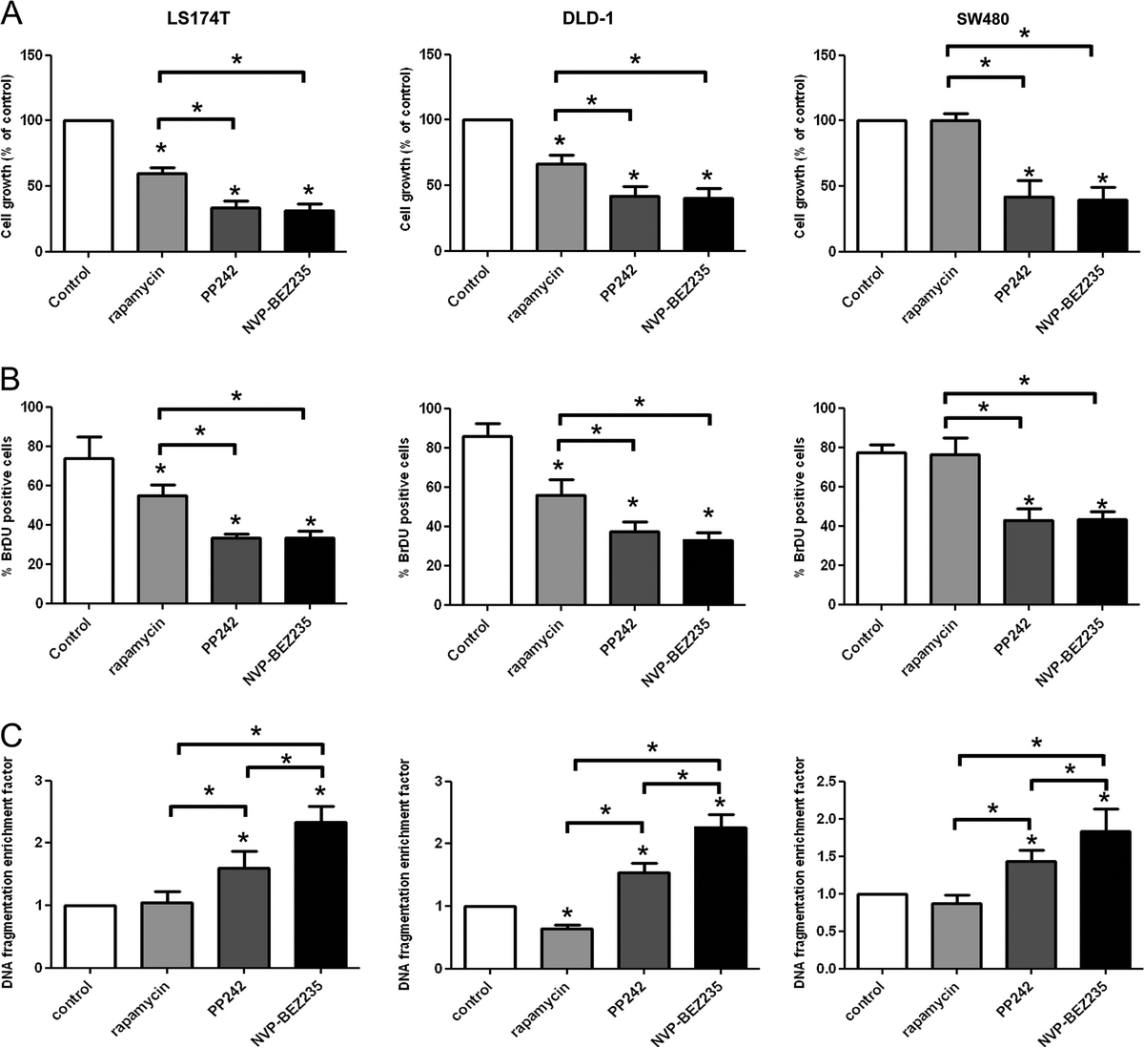
**Comparison of the effects of NVP-BEZ235 versus PP242 versus rapamycin on colon cancer cell growth, proliferation and apoptosis**. LS174T, DLD-1 and SW480 colon cancer cells were treated with 10 nM of rapamycin, 100 nM of NVP-BEZ235 or 100 nM of PP242 for 48 hours. A: Cell growth was determined using a colorimetric MTS assay. Columns, mean cell growth relative to control of three independent experiments; bars, SD. B: Cell proliferation was assessed by BrDU incorporation. Results are quantified as percentage of positive cells for BrDU incorporation. Columns, mean percentage of three independent experiments; bars, SD C: Cell apoptosis was evaluated using a cell death detection ELISA. Cells were harvested and apoptosis was measured by quantifying DNA fragmentation. Columns, mean enrichment factor at 405 nm of three independent experiments; bars, SD. *, *P *< 0.05, compared to control or otherwise as specified by brackets.

To evaluate the activity of rapamycin, NVP-BEZ235 and PP242 on tumor cell growth, colon cancer cell lines were treated for 48 hours and cell growth was analyzed by MTS assay. We found that NVP-BEZ235 and PP242 significantly reduced LS174T, DLD-1 and SW480 cell growth (Figure 2A). Rapamycin also reduced cell growth of LS174T and DLD-1 cells but to a lesser extent than PP242 or NVP-BEZ235. Rapamycin had no effect on SW480 cells (Figure 2A). In addition, NVP-BEZ235 and PP242 also significantly reduced tumor growth of a larger panel of colon cancer cell lines including SW620 and Caco-2 cells (all *PI3KCA *wild type) as well as HT-29 and HCT-116 (*PI3KCA *mutated) [23]. Rapamycin had no effect on Caco-2 and SW620 cells and reduced the growth of HT29 and HCT-116 cells (Additional File 1).

To next investigate whether the effects induced by mTOR inhibitors on colon cancer cell growth result from a reduction of cell proliferation, we performed 5-bromo-2'-deoxyuridine (BrDU) incorporation assay. NVP-BEZ235 and PP242 significantly decreased BrDU incorporation in colon cancer cell lines. Similarly to what we observed on cell growth, rapamycin decreased BrDU incorporation in LS174T and DLD-1 cells but not in SW480 cells (Figure 2B). Finally, we also investigated whether mTOR inhibitors induce apoptosis of colon cancer cells by using a cell death detection ELISA. We observed that NVP-BEZ235 and PP242 increased colon cancer cell apoptosis in all cell lines tested. The effect of NVP-BEZ235 was significantly stronger than PP242. In contrast, rapamycin failed to induce colon cancer cell apoptosis in LS174T and SW480 cells and significantly reduced apoptosis in DLD-1 cells (Figure 2C). Similar results were obtained by quantifying the apoptotic population of colon cancer cells following treatments using propidium iodide staining and flow cytometry analysis (Additional File 2). Taken together, these results show that ATP-competitive inhibitors of mTOR reduce colon cancer cell proliferation and survival.

### ATP-competitive inhibitors of mTOR reduce the growth of colon cancer xenografts

**Figure 3:**
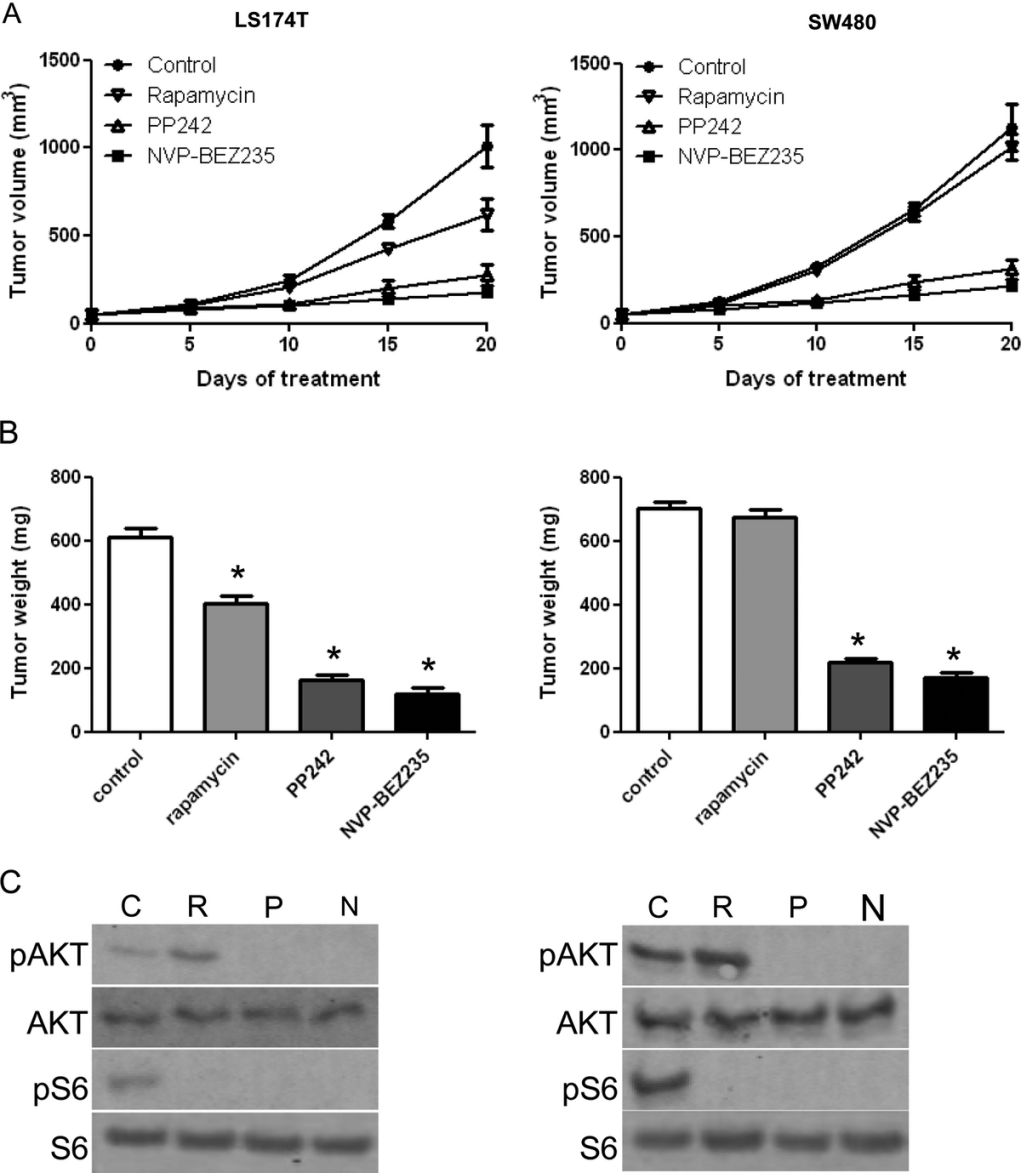
**Effect of Rapamycin, NVP-BEZ235 and PP242 on the growth of LS174T and SW480 tumor xenografts**. **A**: An equal amount of LS174T (left panel) or SW480 (right panel) cells were harvested and injected subcutaneously into nude mice. Once the tumor reached 25 mm^3 ^mice were randomized into four groups and treatments were started with vehicle (Control), rapamycin (R, 1.5 mg/kg/day), NVP-BEZ235 (N, 30 mg/kg/day) or PP242 (P, 60 mg/kg/day). Five mice were included in each group. Tumor volumes were evaluated using caliper measurements and calculated with the formula V = π/6 × *a*^2 ^× *b *where *a *is the short axis and *b *the long axis of the tumor. Points, mean value of tumor volume; bars, SD. **B**: After 20 days of treatment, mice were sacrificed, tumor xenografts were harvested and tumor weight was measured. Columns, mean tumor weight (five tumor xenografts in each group); bars, SD. *, *P *< 0.05, compared to control. **C**: Tumor xenografts were lysed and analyzed by Western blot for phospho-S6 ribosomal protein, total S6 ribosomal protein, phospho-Akt and total Akt. The illustrated blots are representative of three independent experiments.

To evaluate the anticancer effects of mTOR inhibitors *in vivo*, nude mice bearing established LS174T or SW480 tumor cell xenografts were treated with rapamycin, NVP-BEZ235 or PP242 and tumor growth was monitored and compared between each treatment. Rapamycin, NVP-BEZ235 and PP242 reduced the growth of LS174T tumor xenografts (Figure 3A, B). NVP-BEZ235 and PP242 also slowed the growth of SW480 xenografts. In contrast, rapamycin had no effect. Nude mice were administered once a day with rapamycin, NVP-BEZ235 or PP242 at doses that were effective in blocking mTORC1 and mTORC2 as assessed by Western blot analysis of tumor lysates (Figure 3C).

### Effect of ATP-competitive inhibitors of mTOR in combination with U0126 on colon cancer cell growth

**Figure 4:**
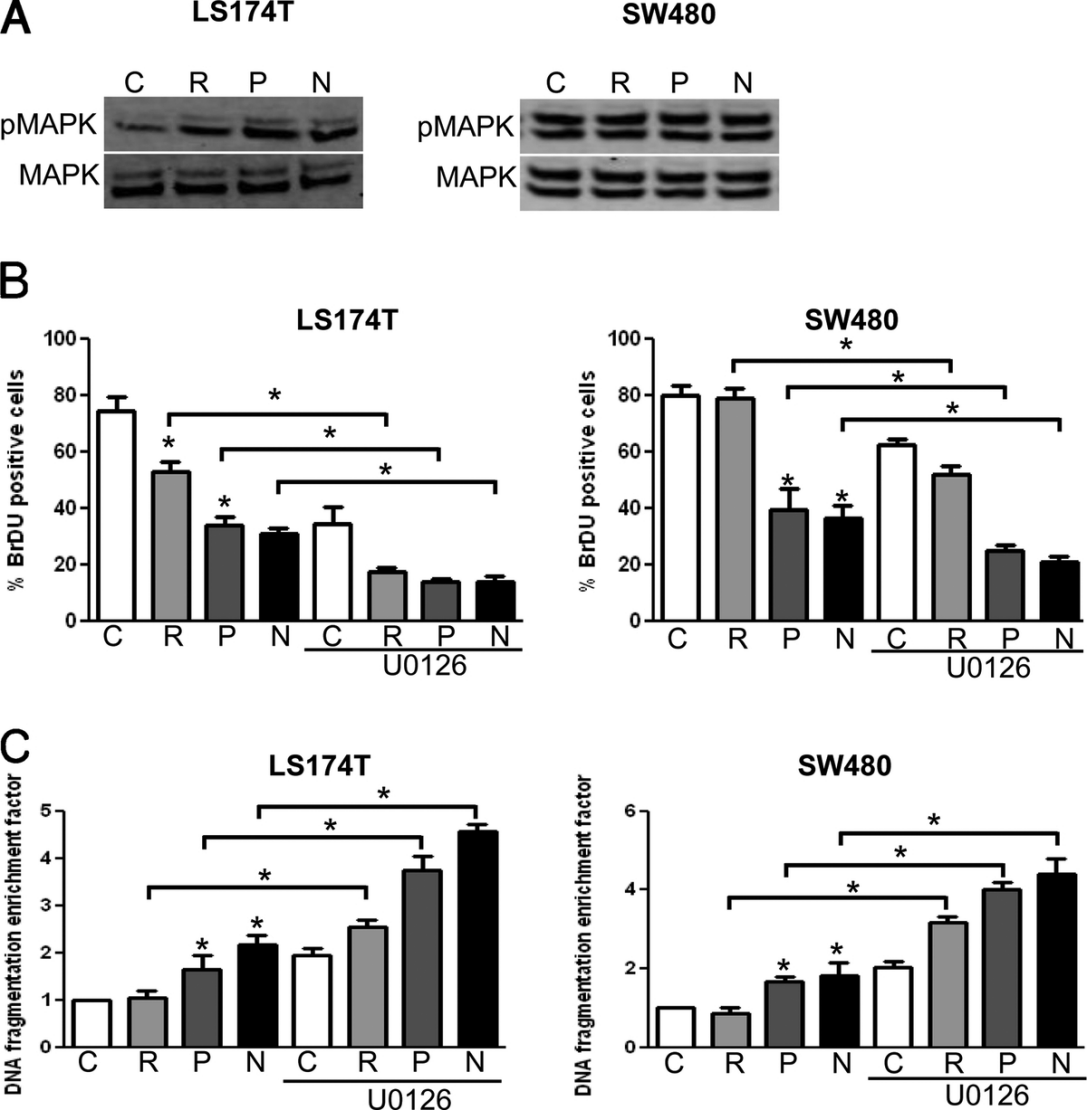
**U0126, a MEK inhibitor, potentiates the anticancer efficacy of ATP-competitive inhibitors of mTOR *in vitro***. **A: **LS174T cells (left panel) or SW480 cells (right panel) were treated with rapamycin (R, 10 nM), NVP-BEZ235 (N, 100 nM), PP242 (P, 100 nM) or DMSO as a control (C) for 4 hours. Cells were subsequently lysed and lysates were analyzed by Western Blot for phospho-MAPK and MAPK. The illustrated blots are representative of three independent experiments. **B: **LS174T and SW480 colon cancer cells were treated with rapamycin (R, 10 nM), NVP-BEZ235 (N, 100 nM) or PP242 (P, 100 nM) for 48 hours in combination or not with U0126 (10 μM). Cell proliferation was assessed by BrDU incorporation. Results are quantified as percentage of positive cells for BrdU incorporation. Columns, mean percentage of three independent experiments; bars, SD **C**: Cells were processed as under panel B and cell apoptosis was evaluated using a cell death detection ELISA. Cells were harvested and apoptosis was measured by quantifying DNA fragmentation. Columns, mean enrichment factor at 405 nm of three independent experiments; bars, SD.

Several studies have shown that the use of mTOR inhibitors induces the activation of MEK/MAPK signaling pathway which reduces the anticancer effects of mTOR inhibitors [26,27]. To test whether the inhibition of mTOR induces MEK/MAPK activation in colon cancer cells, LS174T and SW480 cells were treated with rapamycin, PP242 or NVP-BEZ235 and the phosphorylation of MAPK was assessed by Western blot. We found that rapamycin, PP242 and NVP-BEZ235 increased MAPK phosphorylation in LS174T cells but not in SW480 cells (Figure 4A). To next address whether targeting MEK/MAPK signaling pathway would enhance the anticancer activity of mTOR inhibitors, we treated LS174T and SW480 colon cancer cells with U0126 [28], a MEK inhibitor, in combination or not with mTOR inhibitors. We observed that U0126 potentiated the anti-proliferative and proapoptotic effects of NVP-BEZ235 and PP242 in both cell lines tested (Figure 4B, C).

**Figure 5:**
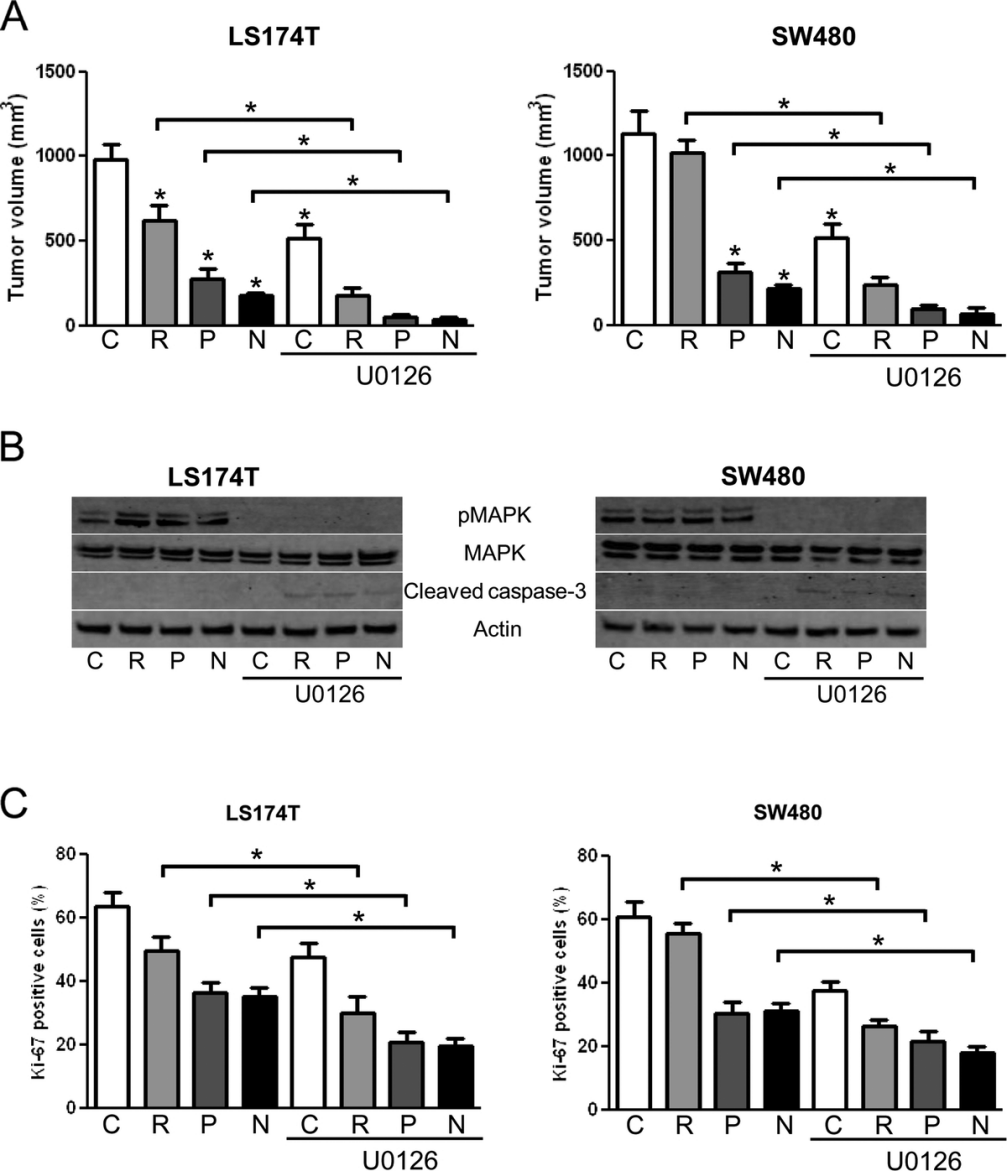
**Antitumoral activity of ATP-competitive inhibitors of mTOR combined with U0126**. A: Nude mice bearing LS174T (left panel) or SW480 (right panel) tumor xenografts were treated with vehicle (C), rapamycin (R, 1.5 mg/kg/day), PP242 (P, 60 mg/kg/day), NVP-BEZ235 (N, 30 mg/kg/day), in combination or not with U0126 (40 μmol/kg/d, i.p.). After 20 days of treatment, tumor volumes were evaluated using caliper measurements and calculated with the formula V = π/6 × *a*^2 ^× *b *where *a *is the short axis and *b *the long axis of the tumor. Columns, mean tumor volume (five tumor xenografts in each group); bars, SD. *, *P *< 0.05, compared to control, or otherwise as specified by brackets. B: Tumor lysates were generated from the harvested xenografts and analyzed for pMAPK, MAPK, cleaved caspase-3 and actin expression. C: Frozen sections of the harvested tumor xenografts were stained with an anti-Ki-67 antibody. The effect of the different treatments on Ki-67 positivity was quantified and expressed as % of cells positive for Ki-67/total number of cells (300 cells counted per tumor; five tumors in each group). Columns, mean % of cells positive for Ki-67 staining; bars, SD. *, *P *< 0.05 compared to control, or otherwise as specified by brackets.

Similarly, *in vivo*, the growth of LS174T or SW480 xenografts was significantly reduced when mice were treated with rapamycin, PP242 or NVP-BEZ235 in combination with U0126 compared to either treatment alone (Figure 5A). Western blot analysis of the tumor lysates showed that, as observed *in vitro*, mTOR inhibitors increased MAPK phosphorylation in LS174T but not in SW480 xenografts. As expected, MAPK phosphorylation was inhibited by U0126 (Figure 5B). The analysis of the tumors subjected to each treatment revealed that ATP-competitive inhibitors of mTOR and U0126 reduced tumor cell proliferation as evidenced by decreased levels of Ki-67 staining. The anti-proliferative effects was increased when mTOR inhibitors were used in combination with U0126 (Figure 5C). In addition, Western blot analysis also showed that combining mTOR inhibitors with U0126 resulted in expression of cleaved caspase-3 which was not observed when mTOR inhibitors and U0126 were used alone (Figure 5B). Taken together, these results show that the concomitant pharmacological blockade of MEK enhances the anticancer activity of mTOR inhibitors. They also suggest that mTOR inhibitors exert a stronger anti-proliferative effect and induce apoptosis when used in combination with U0126.

## Discussion

mTOR represents a promising target in colon cancer. Indeed, components of mTOR signaling pathways are frequently over-expressed and activated in human samples of colon cancer [10,11]. In addition, in experimental settings, the inhibition of mTOR components using siRNA or shRNA results in a marked reduction of colon cancer cell growth in vitro and tumor xenograft growth in vivo [10,11,14]. Furthermore, in a transgenic mouse model in which the adenomatous polyposis coli tumor suppressor gene has been mutated, the inhibition of mTORC1 by the rapamycin analog everolimus, decreased the formation of intestinal polyps and reduced mortality of these mice [15].

Initial studies used rapalogs to target mTOR. However, recent findings have demonstrated that targeting mTOR signaling pathway with rapalogs might not be optimal [16]. In fact, rapalogs block only certain functions of mTORC1 and have no effects on mTORC2. Moreover, the inhibition of mTORC1 by rapalogs also results in the activation of proliferative and survival signals such as the PI3K/Akt and MEK/MAPK signaling pathways through the removal of a negative feedback loop [25]. To overcome these limitations, a new class of mTOR inhibitors has been developed that block the kinase domain of mTOR and therefore inhibit both mTORC1 and mTORC2 [16,29]. In this study, we found that two such inhibitors, PP242, a specific inhibitor of mTOR and NVP-BEZ235, a dual PI3K/mTOR inhibitor, effectively reduced colon cancer cell proliferation and survival and the growth of colon cancer tumor xenografts. Consistent with our findings, a recent study also demonstrated the efficacy of NVP-BEZ235 in a genetically engineered mouse model of CRC [30]. Therefore our results provide rationale for the clinical evaluation of ATP-competitive inhibitors of mTOR in colon cancer patients.

We initially hypothesized that ATP-competitive inhibitors of mTOR would produce anticancer activity only in cells harboring *PI3KCA *mutations. To support this hypothesis it was previously reported that NVP-BEZ235 was effective in PI3K but not in KRAS mutated breast cancer cells and similar findings were reported in a murine model of lung cancer [31,32]. However, we observed here that ATP-competitive inhibitors of mTOR exhibited anticancer effects on both *PI3KCA *mutated as well as on *PI3KCA *wild type colon cancer cells. Consistent with our findings, NVP-BEZ235 is effective in a mouse model of sporadic *PI3KCA *wild type CRC suggesting that the antitumor activity of ATP-competitive inhibitors of mTOR is not restricted to *PI3KCA *mutated colon cancer cells [30].

The anticancer efficacy of NVP-BEZ235 and PP242 was both *in vitro *and *in vivo *superior to rapamycin. It is however worth noting that despite blocking mTORC1 activity *in vivo*, the doses of rapamycin that we used (1.5 mg/kg/day) were lower than those reported by other groups (5 mg/kg/day and 20 mg/kg/day) [33,34]. Therefore a comparison between ATP-competitive inhibitors of mTOR and higher concentrations of rapamycin is needed to conclude that ATP-competitive inhibitors of mTOR are more efficient than rapamycin. Nevertheless, similar to what we found, it was reported in renal cell carcinoma, that the anticancer efficacy of NVP-BEZ235 was superior to rapamycin used at 3.5 mg/kg/day [35].

Our findings also suggest that ATP-competitive inhibitors of mTOR display a broader anticancer activity than rapalogs. We found that while rapamycin had no effect on SW480 colon cancer cells, PP242 and NVP-BEZ235 reduced SW480 cell proliferation and survival as well as the growth of SW480 xenografts. Similarly, it was reported that blocking mTORC1 by rapamycin or by the use of raptor siRNA had no effect on the proliferation of SW480 cells. In contrast, targeting mTORC2 with rictor siRNA efficiently reduced SW480 cell proliferation [10]. Therefore, by blocking mTORC2 in addition to mTORC1, the anticancer activity of ATP-competitive inhibitors of mTOR appear to be broader than rapamycin.

Emerging evidence has shown that blocking mTORC1 results in the removal of a negative feedback loop resulting in the activation of the PI3K/Akt and MEK/MAPK signaling pathways that counteract the anticancer efficacy of mTOR inhibitors [25]. In our study, we observed that ATP-competitive inhibitors of mTOR increased MAPK phosphorylation in LS174T cells (Figure 4a). Similar effects were reported in other cell types including renal cancer cells, Waldenstrom macroglobulinemia cells, sarcoma cells and endothelial cells [35-38]. We further observed that targeting MAPK with a MEK inhibitor in combination with mTOR inhibitors resulted in synergistic inhibition of LS174T and SW480 colon cancer cell growth (Figure 4b-d). Noteworthy, we found that ATP-competitive inhibitors of mTOR did not increase MAPK phosphorylation in SW480 suggesting that MEK inhibitors would potentiate the anticancer efficacy of mTOR inhibitors regardless of whether mTOR inhibitors increase MAPK phosphorylation.

## Conclusions

Overall, our study shows that ATP-competitive inhibitors of mTOR efficiently reduced the growth of colon cancer cells both *in vitro *and *in vivo*. In addition, it also shows that the anticancer efficacy of ATP-competitive inhibitors of mTOR is potentiated by the simultaneous pharmacological blockade of the MEK/MAPK signaling pathway in colon cancer cells. Therefore, ATP-competitive inhibitors represent promising agents in the treatment of CRC that warrant to be tested in clinical trials.

## Abbreviations

mTOR: Mammalian target of rapamycin; PI3K: Phosphatidylinositol 3-kinase; CRC: Colorectal cancer; BrDU: 5-bromo-2'-deoxyuridine; PI3KCA: Catalytic subunit of phosphatidylinositol 3-kinase; DMSO: Dimethyl sulfoxide; PBS: Phosphate buffered saline.

## Competing interests

The authors declare that they have no competing interests.

## Authors' contributions

BB and OD designed the study. BB, LW, ADM, MD, DR, OD performed the experiments and interpreted the experimental findings. BB drafted the manuscript. ND and OD wrote the final version of the manuscript. All authors read and approved the final manuscript.

## Pre-publication history

The pre-publication history for this paper can be accessed here:

http://www.biomedcentral.com/1471-2407/12/86/prepub

## Supplementary Material

Additional file 1 **Effects of rapamycin, PP242 and NVP-BEZ235 on the growth of Caco-2, SW620, HT29 and HCT-116 colon cancer cells**. Caco-2, SW620, HT29 and HCT-116 colon cancer cells were treated with 10 nM of rapamycin, 100 nM of PP242, 100 nM of NVP-BEZ235 or DMSO as a control for 48 hours. Cell growth was determined using a colorimetric MTS assay. Columns, mean cell growth relative to control of three independent experiments; bars, SD. *P *< 0.05, compared to control or otherwise as specified by brackets. Click here for file

Additional file 2 **Effects of rapamycin, PP242 and NVP-BEZ235 on the growth of Caco-2, SW620, HT29 and HCT-116 colon cancer cells**. Caco-2, SW620, HT29 and HCT-116 colon cancer cells were treated with 10 nM of rapamycin, 100 nM of PP242, 100 nM of NVP-BEZ235 or DMSO as a control for 48 hours. Cell growth was determined using a colorimetric MTS assay. Columns, mean cell growth relative to control of three independent experiments; bars, SD. *P *< 0.05, compared to control or otherwise as specified by brackets. Click here for file
