# Supplementary figures and images for: Antitumor activities of ATP-competitive inhibitors of mTOR in colon cancer cells
Source: BMC Cancer. 2012 Mar 8;12:86. doi: 10.1186/1471-2407-12-86 (PMC3314574; doi:10.1186/1471-2407-12-86)

## Slide 1
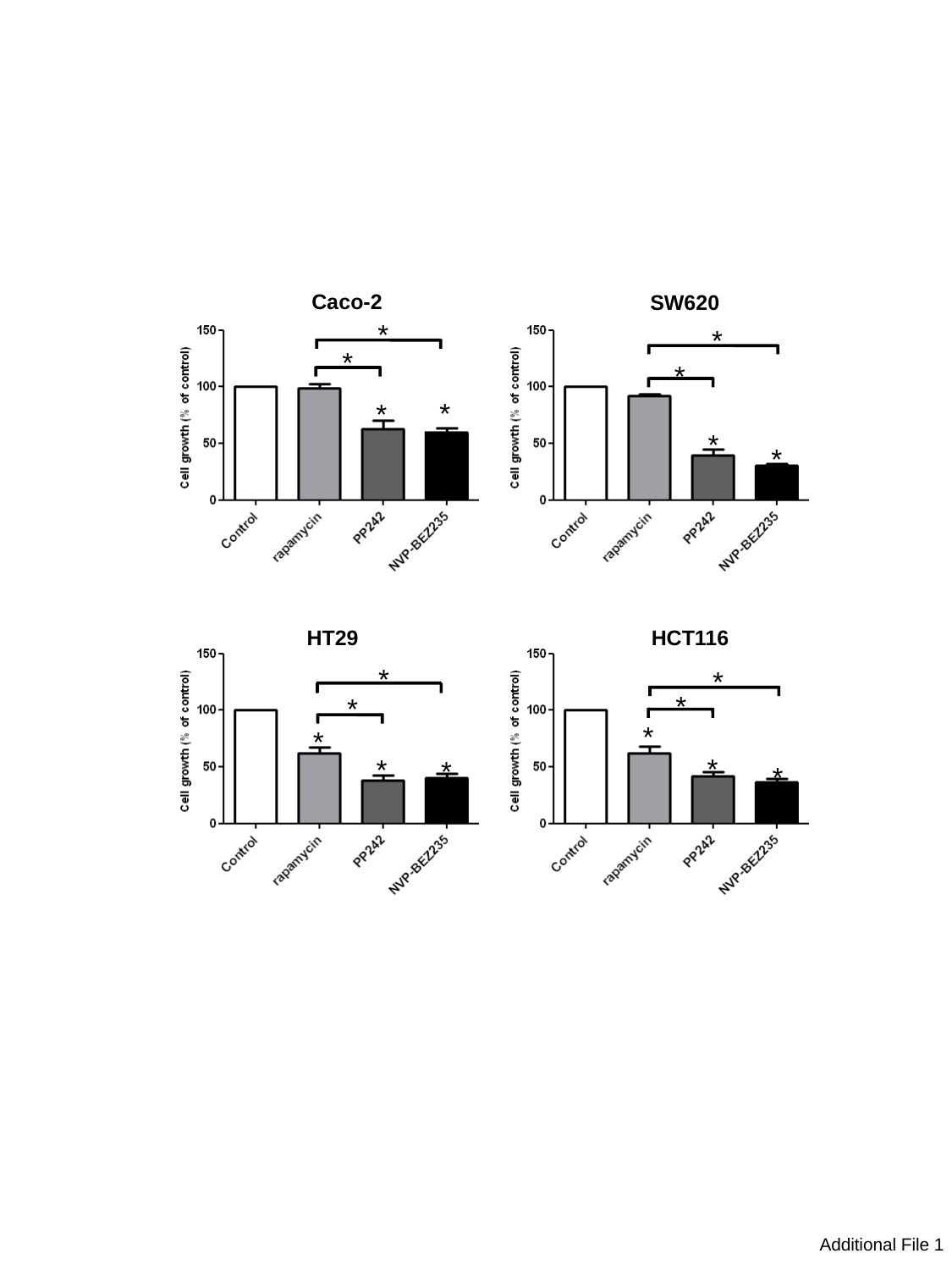

Caco-2
SW620
*
*
*
*
*
*
*
*
*
HT29
HCT116
*
*
*
*
*
*
*
*
*
*
Additional File 1

Supplement: Additional file 1 — Effects of rapamycin, PP242 and NVP-BEZ235 on the growth of Caco-2, SW620, HT29 and HCT-116 colon cancer cells. Caco-2, SW620, HT29 and HCT-116 colon cancer cells were treated with 10 nM of rapamycin, 100 nM of PP242, 100 nM of NVP-BEZ235 or DMSO as a control for 48 hours. Cell growth was determined using a colorimetric MTS assay. Columns, mean cell growth relative to control of three independent experiments; bars, SD. P < 0.05, compared to control or otherwise as specified by brackets. [file 1471-2407-12-86-S1.PPT]

## Slide 1
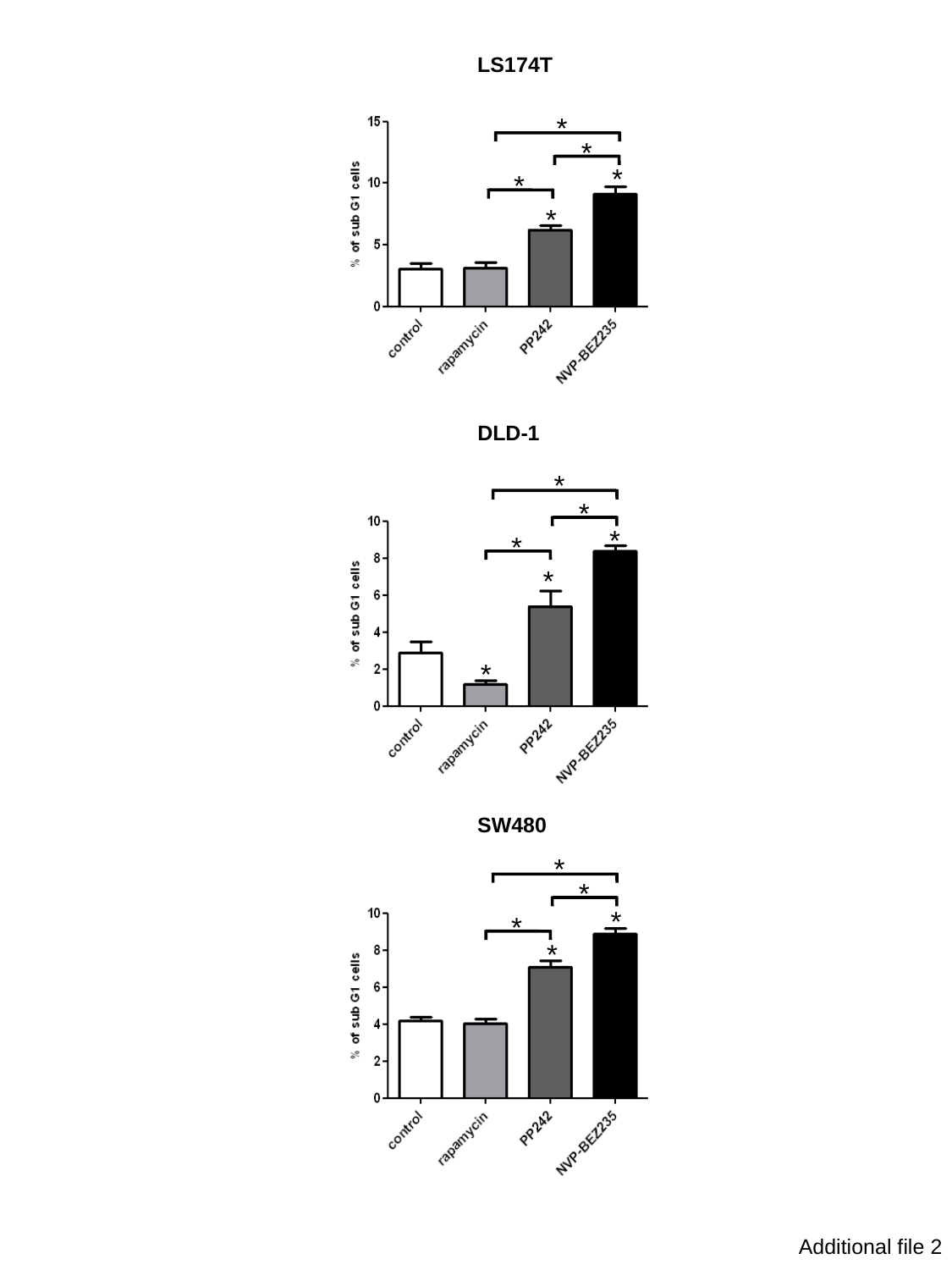

LS174T
*
*
*
*
*
DLD-1
*
*
*
*
*
*
SW480
*
*
*
*
*
Additional file 2

Supplement: Additional file 2 — Effects of rapamycin, PP242 and NVP-BEZ235 on the growth of Caco-2, SW620, HT29 and HCT-116 colon cancer cells. Caco-2, SW620, HT29 and HCT-116 colon cancer cells were treated with 10 nM of rapamycin, 100 nM of PP242, 100 nM of NVP-BEZ235 or DMSO as a control for 48 hours. Cell growth was determined using a colorimetric MTS assay. Columns, mean cell growth relative to control of three independent experiments; bars, SD. P < 0.05, compared to control or otherwise as specified by brackets. [file 1471-2407-12-86-S2.PPT]
